# Effects of long-term PM_**2.5**_ exposure on metabolic syndrome among adults and elderly in Guangdong, China

**DOI:** 10.1186/s12940-022-00888-2

**Published:** 2022-09-10

**Authors:** Xue-yan Zheng, Si-li Tang, Tao Liu, Ye Wang, Xiao-jun Xu, Ni Xiao, Chuan Li, Yan-jun Xu, Zhao-xuan He, Shu-li Ma, Yu-liang Chen, Rui-lin Meng, Li-feng Lin

**Affiliations:** 1 Guangdong Provincial Center for Disease Control and Prevention, Guangdong, China; 2 School of Public Health, Southern Medical University, Guangzhou, China; 3 Disease Control and Prevention Institute of Jinan University, Jinan University, Guangzhou, China; 4 Department of Epidemiology and Biostatistics, School of Public Health, Guangdong Pharmaceutical University, Guangzhou, China

**Keywords:** PM_2.5_, Metabolic syndrome, Blood pressure, Triglyceride, High-density lipoprotein cholesterol, Fasting blood glucose, Waist circumference

## Abstract

**Background:**

We aimed to explore the association between long-term exposure to particulate matter ≤ 2.5 µm (PM_2.5_) and metabolic syndrome (MetS) and its components including fasting blood glucose (FBG), blood pressure, triglyceride (TG), high-density lipoprotein cholesterol (HDL-c) and waist circumference among adults and elderly in south China.

**Methods:**

We surveyed 6628 participants in the chronic disease and risk factors surveillance conducted in 14 districts of Guangdong province in 2015. MetS was defined based on the recommendation by the Joint Interim Societies’ criteria. We used the spatiotemporal land-use regression (LUR) model to estimate the two-year average exposure of ambient air pollutants (PM_2.5_, PM_10_, SO_2_, NO_2_, and O_3_) at individual levels. We recorded other covariates by using a structured questionnaire. Generalized linear mixed model was used for analysis.

**Results:**

A 10-μg/m^3^ increase in the two-year mean PM_2.5_ exposure was associated with a higher risk of developing MetS [odd ratio (OR): 1.17, 95% confidence interval (CI): 1.01, 1.35], increased risk of fasting blood glucose level.

(OR: 1.18, 95% CI: 1.02, 1.36), and hypertriglyceridemia (OR: 1.36, 95% CI: 1.18, 1.58) in the adjusted/unadjusted models (all *P* < 0.05). We found significant interaction between PM_2.5_ and the region, exercise on the high TG levels, and an interaction with the region, age, exercise and grain consumption on FBG (*P*_interaction_ < 0.05).

**Conclusions:**

Long-term exposure to PM_2.5_ was associated with MetS, dyslipidemia and FBG impairment. Efforts should be made for environment improvement to reduce the burden of MetS-associated non-communicable disease.

**Supplementary Information:**

The online version contains supplementary material available at 10.1186/s12940-022-00888-2.

## Introduction

Metabolic syndrome (MetS) is a cluster of metabolic disorders including abdominal obesity, hypertension, hypertriglyceridemia, low high-density lipoprotein cholesterol (HDL-c) and hyperglycemia [1]. MetS has been recognized as an urgent public health concern because it affects 20–30% of the global population, of which the standardized prevalence of MetS is around 24.2% in China [2, 3]. Previous studies showed that MetS was associated with an increased risk of cardiovascular diseases (CVDs), diabetes mellitus, cancers and other chronic non-communicable diseases [4, 5]. Evidence suggests that MetS-related adverse health outcomes may be enhanced not only by genetic factors, physical inactivity and unhealthy diet [6–9], but also by environmental pollutant exposure [10, 11], including air pollution.

Accumulating studies have added to the evidence that the inhalation of particulate matte ≤ 2.5 µm (PM_2.5_) might lead to pulmonary oxidative stress, systemic inflammation, vascular dysfunction and atherosclerosis [12–16]. Previous studies suggested that PM_2.5_ was the major risk factor for adverse health outcomes including hypertension [12], obesity [13], elevated fasting blood glucose (FBG) [14, 15], waist circumference [16] and dyslipidemia [17], which were crucial components in the diagnosis of MetS. However, the effects of PM_2.5_ on blood pressure [18, 19], fasting blood glucose [20, 21] and obesity [22–24] still remained inconsistent. Furthermore, the evidence concerning the associations of air pollution and MetS is still scarce. To our knowledge, only a few studies have reported the detrimental effects of long-term exposure to ambient air pollution on MetS [17, 25–28], which were mainly conducted in the developed countries such as Korea, North America or Saudi Arabia [17, 25, 26]. Only two epidemiological studies evaluated the associations between PM_2.5_ and the prevalence of MetS in the developing countries such as China [27, 28] among adolescents and children [27], and adults and elderly [28]. In addition, the effects of PM_2.5_ on specific components on MetS in Chinese population was limited based on the prior evidence.

As one of the most developed provinces in southern China, there has been considerable lifestyle and dietary changes during these decades in Guangdong, resulting in the increase of MetS and stroke, coronary heart disease, and cancers [29]. Meanwhile, air pollution has become one of the most severe environmental problem in Guangdong [30]. In the CAPES study, despite a relatively low concentrations of PM, there was a higher risk of the total, cardiovascular and respiratory mortality attributed to PM in Guangzhou (the capital city of Guangdong province), compared with the heavy industry cities in northeastern China, where PM pollution was more severe [31]. The relatively higher concentration of the toxic component including polybrominated diphenyl ethers (PBDEs) found in PM_2.5_ in southern China [32, 33] might help provide the evidence for the stronger association between PM and mortality.

Considering the current MetS epidemic, the more toxic effect of PM_2.5_ in south China, the inconsistent effects of PM_2.5_ on specific components of MetS, and the limited information of the association between PM_2.5_ and MetS, we explored the effects of ambient PM_2.5_ pollution on MetS and its components [blood pressure, triglyceride (TG), high-density lipoprotein-cholecsterol (HDL-c), fasting blood glucose (FBG) and waist circumference] in Guangdong, China. To address the knowledge gap, our findings would provide important public health implications which aimed to reduce the detrimental impact of ambient air pollution of PM_2.5_ on CVDs and MetS in China.

## Materials and methods

### Study design and participants

This study was conducted using a multistage, probability-based sampling strategy, based on the *Chronic Disease and Risk Factors Surveillance* in 2015 in Guangdong province, China. 14 surveillance points were randomly selected. Between October 2015 and February 2016, adults aged 18 years who were living in the current residence for at least 6 months were recruited. All participants were interviewed face-to-face by using a structured questionnaire, which has been described previously [34, 35]. In addition, participants underwent anthropometric measurements (blood pressure, fasting glucose, blood pressure, waist circumstance, height and weight) and blood sample collection by the well-trained public health practitioners from the local health stations or community health service centers. The study protocol was approved by the ethics review committee of the National Center for Chronic and Non-Communicable Disease Control and Prevention, China Center for Disease Control and Prevention. All participants were provided written informed consent. Inclusion and exclusion criteria of participants have been reported previously [36].

### MetS definition

The diagnosis of MetS [1] was based on the Joint Interim Societies’ definition. Participants were considered to have MetS if they met any three of the five following conditions (1): Elevated TG levels: ≥ 1.7 mmol/l (150 mg/dl) [1]; (2) Decreased HDL-c levels: < 1.0 mmol/l (40 mg/dl) for men; < 1.3 mmol/l (50 mg/dl) for women [1]; (3) Elevated blood pressure [systolic blood pressure (SBP) ≥ 130 or diastolic blood pressure (DBP) ≥ 85 mmHg] [1]; (4) Elevated FBG levels [FBG ≥ 5.6 mmol/L (100 mg/dl)] [1]; (5) Central obesity, defined as an elevated waist circumference according to the WHO criteria: ≥ 90 cm for men; ≥ 80 cm for women [37]. See Table 1 for further details.

**Table 1:** Criteria for clinical diagnosis of the metabolic syndrome

Conditions	Recommended threshold	
	**For Men**	**For women**
Elevated TG levels	≥ 1.7 mmol/l (150 mg/dl)	≥ 1.7 mmol/l (150 mg/dl)
Decreased HDL-c levels	< 1.0 mmol/l (40 mg/dl) for males	< 1.3 mmol/l (50 mg/dl)
Elevated blood pressure	Elevated blood pressure	Elevated blood pressure
Elevated FBG levels	FBG ≥ 5.6 mmol/l (100 mg/dl)	FBG ≥ 5.6 mmol/l (100 mg/dl)
Central obesity	waist circumference ≥ 90 cm	waist circumference ≥ 80 cm

Participants were considered to have MetS if they meet any three of the five following conditions (1): Elevated TG levels: ≥ 1.7 mmol/l (150 mg/dl); (2) Decreased HDL-c levels: < 1.0 mmol/l (40 mg/dl) for men; < 1.3 mmol/l (50 mg/dl) for women; (3) Elevated blood pressure (SBP ≥ 130 or DBP ≥ 85 mmHg); (4) Elevated FBG levels [FBG ≥ 5.6 mmol/l (100 mg/dl)]; (5) Central obesity was defined as elevated waist circumference: ≥ 90 cm for men; ≥ 80 cm for women

### Assessment of long-term exposure to air pollution

We used the spatiotemporal land-use regression (LUR) model to estimate the two-year average exposure of ambient air pollutants including PM_2.5_, particulate matter < 10 µm (PM_10_), sulfur dioxide (SO_2_), nitrogen dioxide (NO_2_) and ozone (O_3_) at individual levels. The details of the data and prediction process has been published previously [38], which were as follows:

1. The spatiotemporal LUR model was built with the following predictors: population density, road length, land-use data (farmland, blue space, living land, and green space), and ambient visibility. Two smooth temporal basis functions were analyzed to estimate the secular trend of air pollution. The R^2^ was 88.86% with the root mean square error (RMSE) of 5.65%, based on the findings of the tenfold cross-validation.
2. Residence address was extracted from the questionnaire and included into the model to forecast the weekly average air pollution between April 2013 and December 2016.
3. The two-year averaged air pollutant concentrations before the investigation date were estimated for each individual.

### Covariates

The following covariates were incorporated to examine the potential confounding and mediating effects: age, sex (man and woman), race (Han and minority), region (urban and rural), occupation (physical work and non-physical work), education level (none, primary school education, middle school education, university education or higher), marital status (none, primary school education, middle school education and university education or higher), household income (< 30, 30–50, 50–100, 100–200 and ≥ 200 × 1000 RMB), weight change in the past year (an increase of > 2.5 kg, unchanged < 2.5 kg, a decease of > 2.5 kg and unclear), alcohol consumption, exercise, family history of diabetes (no and yes), exercise (no and yes), alcohol consumption (no and yes), passive smoking (no and yes), cigarette smoking (non-smoker and smoker), biomass fuel use (no and yes), body-mass index (BMI) (under weight, normal and overweight/obese), grain consumption, vegetable and fruit consumption and red meat consumption. The definition of the covariates is summerized in E-Table 1 [34, 35, 39, 40].

### Statistical analysis

We analyzed the characteristics between the groups with MetS and without MetS, by demonstrating the mean and standard deviation for continuous variables and frequencies for categorical variables. The t-test was performed to analyze the distribution of continuous variables, and when indicated, appropriate transformation was applied. A contingency table and Chi-squared test was performed for analyzing the frequencies of categorical variables. The normality and equality of variance was assessed by using the Shapiro–Wilk’s test and Bartlett’s test, respectively. The odds ratios (ORs) and 95% confidence intervals (95%CIs) were calculated for determining the association between ambient air pollutant exposure to PM_2.5_ and the presence of MetS and its components by using the generalized linear mixed model, based on the three stepwise models to confirm the validity of findings. Family was treated as random effect by calculating the intraclass correlation coefficient (ICC). We compared the Akaike's information criterion value of these three models to avoid over-fitting. The magnitude of collinearity was assessed based on the variance inflation factor (VIF). The VIF of 5 or greater indicated collinearity among the variables. Variables with the evidence of a significant collinearity were excluded from the model. The Spearman’s rank correlation test was used to determine the relationship between pollutants. Strong, moderate, and weak correlations were defined as the coefficients (r_s_) greater than 0.60, 0.30 to 0.60, and less than 0.30, respectively. Since strong and moderate correlation was identified between PM_2.5_ and other pollutant models, we only applied the single pollutant model (PM_2.5_) to avoid covariance. We further stratified the study participants by the region, sex, age, cigarette smoking, alcohol consumption, exercise, BMI, grain consumption, vegetable and fruit consumption and red meat consumption, to study the significant associations between PM_2.5_ and MetS, high TG and FBG in each stratum. We also included the interaction terms in the generalized linear mixed effect models to test the interactions between PM_2.5_ and MetS, high TG and FBG in each subgroup. All statistical analyses were performed with R software (version 4.0.2). The threshold of statistical significance for *P* value was set to be 0.05.

## Results

A total of 8991 participants were included in this study, among whom 1157 had missing key variables, 252 had previously been diagnosed as having CVDs, 954 had taken measures to control blood pressure, blood glucose, and lipids. Therefore, 6628 participants were included in the final analysis, with a mean age of 50.1 years. Table 2 shows the demographic characteristics of the participants. 1691 of the participants were diagnosed as having MetS, and 4937 without. Participants with MetS were more likely to have poorer education, lower household income, less exercise and higher BMI as compared with participants without MetS (Table 2).

**Table 2:** Basic characteristics of participants by metabolic syndrome

**Characteristics**	**Total** (*n* = 6628)	**Metabolic Syndrome**		**p**
		**Event **(*n* = 1691)	**Non-Event **(*n* = 4937)	
**Age (year), mean (SD)**	50.12 (14.73)	54.09 (12.83)	48.76 (15.09)	< 0.001^*^
**Sex, n (%)**				< 0.001^*^
** Man**	2955 (44.6)	677 (40.0)	2278 (44.6)	
** Women**	3673 (55.4)	1014 (60.0)	2659 (55.4)	
**Race, n (%)**				0.570
** Han**	6562 (99.0)	1672 (98.9)	4890 (99.0)	
** Minority**	66 (1.0)	19 (1.1)	47 (1.0)	
**Region, n (%)**				0.092
** Urban**	3613 (54.5)	892 (52.7)	2721 (55.1)	
** Rural**	3015 (45.5)	799 (47.3)	2216 (44.9)	
**Occupation, n (%)**				0.273
** Physical work**	5070 (76.5)	1310 (77.5)	3760 (76.2)	
** Non-physical work**	1558 (23.5)	381 (22.5)	1177 (23.8)	
**Educational level, n (%)**				< 0.001^*^
** None**	836 (12.6)	303 (17.9)	533 (10.8)	
** Primary school education**	2246 (33.9)	625 (37.0)	1621 (32.8)	
** Middle school education**	2905 (43.8)	656 (38.8)	2249 (45.6)	
** University education or higher**	641 (9.7)	107 (6.3)	534 (10.8)	
**Marriage status, n (%)**				< 0.001^*^
** Unmarried**	357 (5.4)	46 (2.7)	311 (6.3)	
** Married**	5968 (90.0)	1544 (91.3)	4424 (89.6)	
** Widowed or divorced**	303 (4.6)	101 (6.0)	202 (4.1)	
**Household income (× 1000 RMB)**				0.038^*^
** < 30**	1029 (15.5)	261 (15.4)	768 (15.6)	
** 30 ≤ Household income < 50**	1171 (17.7)	321 (19.0)	850 (17.2)	
** 50 ≤ Household income < 100**	1218 (18.4)	280 (16.6)	938 (19.0)	
** 100 ≤ Household income < 200**	514 (7.8)	122 (7.2)	392 (7.9)	
** ≥ 200**	171 (2.6)	34 (2.0)	137 (2.8)	
** Refuse to answer or don't know**	2525 (38.1)	673 (39.8)	1852 (37.5)	
**Behaviors factors**				
**Cigarette smoking**				0.003^*^
** Nonsmoker**	4428 (66.8)	1180 (69.8)	3248 (65.8)	
** Smoker**	2200 (33.2)	511 (30.2)	1689 (34.2)	
**Alcohol consumption, n (%)**				0.027^*^
** No**	3929 (59.3)	1041 (61.6)	2888 (58.5)	
** Yes**	2699 (40.7)	650 (38.4)	2049 (41.5)	
**Exercise, n (%)**				0.019^*^
** No**	5479 (82.7)	1430 (86.6)	4049 (82.0)	
** Yes**	1149 (17.3)	261 (15.4)	888 (18.0)	
**Family history of diabetes mellitus, n (%)**				0.524
** No**	6222 (93.9)	1582 (93.6)	4640 (94.0)	
** Yes**	406 (6.1)	109 (6.4)	297 (6.0)	
**Weight change in the past 12 months, n (%)**				0.221
** Increase in > 2.5 kg**	609 (9.2)	164 (9.7)	445 (9.0)	
** Unchanged (< 2.5 kg)**	4743 (71.6)	1217 (72.0)	3526 (71.4)	
** Decease in > 2.5 kg**	596 (9.0)	132 (7.8)	464 (9.4)	
** Unclear**	680 (10.3)	178 (10.5)	502 (10.2)	
**Household air pollution exposure**				
**Passive smoking, n (%)**				0.598
** No**	1531 (32.0)	393 (31.2)	1138 (32.3)	
** Yes**	3250 (68.0)	868 (68.8)	2382 (67.7)	
**Biomass fuel**				0.323
** No**	5136 (77.5)	1325 (78.4)	3811 (77.2)	
** Yes**	1492 (22.5)	366 (21.6)	1126 (22.8)	
** Grain consumption(g/daily), medium (IQR)**	400.00 (376.90)	394.39 (371.73)	400.00 (380.00)	0.212
** Vegetable and Fruit consumption (g/daily), medium (IQR)**	308.00 (298.35)	308.00 (293.33)	308.33 (299.00)	0.891
** Red Meat consumption (g/daily), medium (IQR)**	71.43 (95.96)	53.57 (96.29)	80.00 (115.86)	0.019^*^
**Ambient air pollution exposure (μg/m**^**3**^**), mean (SD)**				
** PM**_**2.5**_	37.2 (4.8)	37.4 (4.5)	37.1 (4.8)	0.040^*^
** PM**_**10**_	55.4 (5.0)	55.2 (4.8)	55.5 (5.1)	0.041^*^
** SO**_**2**_	16.1 (3.8)	16.2 (3.9)	16.1 (3.8)	0.227
** NO**_**2**_	26.0 (12.6)	25.3 (11.4)	26.2 (12.9)	0.007^*^
** O**_**3**_	56.2 (6.4)	56.7 (5.9)	56.1 (6.5)	0.005^*^
**Anthropometry**				
** BMI (kg/m2), mean (SD)**	23.04 (3.36)	25.65 (3.21)	22.15 (2.92)	< 0.001^*^
**BMI category, n (%)**				< 0.001^*^
** Under weight**	504 (56.4)	15 (0.9)	489 (9.9)	
** Normal**	3741 (7.6)	504 (29.8)	3237 (65.6)	
** Overweight/ Obese**	2383 (36.0)	1172 (69.3)	1211 (24.5)	
**MetS, n (%)**	1691 (25.5)	1691 (100.0)	-	
**Central obesity, n (%)**	2038 (30.7)	1236 (73.1)	802 (16.2)	< 0.001^*^
**High TG, n (%)**	1379 (20.8)	998 (59.0)	381 (7.7)	< 0.001^*^
**Low HDL-c, n (%)**	2759 (41.6)	1325 (78.4)	1434 (29.0)	< 0.001^*^
**Hypertension, n (%)**	3339 (50.4)	1430 (84.6)	1909 (38.7)	< 0.001^*^
**High FBG, n (%)**	1606 (24.2)	920 (54.4)	686 (13.9)	< 0.001^*^

*BMI* Body-mass index, *FBG* Fasting blood glucose, *HDL-c* High-density lipoprotein cholesterol, *IQR* Inter Quartile Range, *n* Number, *MetS* Metabolic syndrome, *NO*_*2*_ Nitrogen dioxide, *O*_*3*_ Ozone, *PM*_*2.5*_ Particulate matter ≤ 2.5 µm, *PM*_*10*_ Particulate matter < 10 µm, *Red meat* beef, pork, lamb, *SD* Standard deviation, *SO*_*2*_ Sulfur dioxide, *TG* Triglyceride

^∗^:*P* < 0.05

Table 3 demonstrates the descriptive statistics of air pollution concentrations in 14 district surveillance points, as well as their pairwise correlations. The range concentration of PM_2.5_, PM_10_, SO_2_, NO_2_, and O_3_ were 27.99 to 46.96 µg/m^3^, 42.17 to 67.33 µg/m^3^, 9.31 to 22.28 µg/m^3^, 7.94 to 62.68 µg/m^3^, 40.54 to 68.83 µg/m^3^, respectively. The mean concentration of PM_2.5_ and PM_10_ exceeded the World Health Organization (WHO) air quality guidelines,, which respective recommended values was 5 µg/m^3^, 15 µg/m^3^ and 10 µg/m^3^ [41] in the surveillance points in this study. In general, the air pollutants were highly or moderately correlated with each other (r_s_ ranged from -0.35 to 0.75).

**Table 3:** Summary statistics and Spearman correlations of 2-year mean air pollutants

	**Summary statistics**					**Spearman correlation coefficients**				
	**Mean**	**Median**	**Minimum**	**Maximum**	**IQR**	**PM2.5**	**PM10**	**SO2**	**NO2**	**O3**
**PM**_**2.5**_** (μg/m**^**3**^**)**	37.17	38.30	27.99	46.96	8.84	1.00	0.71^*^	0.52^*^	0.60^*^	-0.49^*^
**PM**_**10**_** (μg/m**^**3**^**)**	55.43	55.09	42.17	67.33	7.58		1.00	0.63^*^	0.75^*^	-0.51^*^
**SO**_**2**_** (μg/m**^**3**^**)**	16.12	15.92	9.31	22.28	5.44			1.00	0.37^*^	-0.35^*^
**NO**_**2**_** (μg/m**^**3**^**)**	25.98	23.07	7.94	62.68	18.07				1.00	-0.68^*^
**O**_**3**_** (μg/m**^**3**^**)**	56.23	56.96	40.54	68.83	7.38					1.00

*PM*_*2.5*_ Particulate matter ≤ 2.5 µm, *PM*_*10*_ Particulate matter < 10 µm, *SO*_*2*_ Sulfur dioxide, *NO*_*2*_ Nitrogen dioxide, *O*_*3*_ Ozone

Note: Spearman correlation coefficients, ^*^:*P* < 0.05

Table 4 shows the adjusted odds ratios of metabolic syndrome and its components with 10-μg/m^3^ increase in PM_2.5_. For all participants, PM_2.5_ was positively associated with MetS. Results of the single pollutant model showed that each 10 μg/m^3^ increase in two years of exposure to PM_2.5_ was associated with a 1.17-fold (95% CI: 1.01–1.35) higher risk of MetS (*P* < 0.05, model 3). In the analysis of other components of MetS, each 10 μg/m^3^ increase in the two-year mean exposure of PM_2.5_ was associated with high TG and high FBG, with a respective odd ratio (OR) of 1.36 (95% CI: 1.18–1.58) and 1.18 (95% CI: 1.02–1.36) in the single-pollutant model (*P* < 0.05, model 3). No association was observed between ambient PM_2.5_ exposure and central obesity, low HDL-c, and hypertension. Results in model 1 to model 3 were not changed materially, suggesting that the results were robust (Table 4).

**Table 4:** Adjusted odd ratios of metabolic syndrome and its components in overall population with 10-μg/m^3^ increase in PM_2.5_

Variables	MetS				Central obesity				High TG		
	**AIC**	**OR (95%CI)**	***P***		**AIC**		**OR (95%CI)**	***P***	**AIC**	**OR (95%CI)**	***P***
Model 1	7522.5	1.14(1.01, 1.29)	0.039*		8181.9		1.04 (0.93, 1.16)	0.516	6757.9	1.36 (1.34, 1.38)	< 0.001*
Model 2	5807.8	1.17(1.15, 1.19)	< 0.001*		4075.2		1.02 (0.85, 1.23)	0.806	6190.5	1.40 (1.21, 1.62)	< 0.001*
Model 3	5807.8	1.17(1.01, 1.35)	0.042*		4060.1		0.98 (0.82, 1.18)	0.813	6183.0	1.36 (1.18, 1.58)	< 0.001*
**Variables**	**Low HDL-c**			**Hypertension**					**High FBG**		
	**AIC**	**OR (95%CI)**	***P***	**AIC**		**OR (95%CI)**		***P***	**AIC**	**OR (95%CI)**	***P***
Model 1	8989.0	1.00 (0.90, 1.12)	0.944	9171.0		1.04 (0.93, 1.16)		0.506	7302.1	1.17 (1.02, 1.35)	0.023*
Model 2	8358.3	0.98 (0.87, 1.11)	0.784	7680.7		1.02 (0.90, 1.16)		0.721	6871.9	1.15 (1.01, 1.33)	0.047*
Model 3	8318.3	0.99 (0.88, 1.12)	0.867	7658.5		1.03 (0.91,1.16)		0.682	6823.2	1.18 (1.02, 1.36)	0.026*

*Model 1: Exposure to PM*_*2.5*_*;*

*Model 2: Model 1 adjusted with age, sex, education, marital status, body mass index, household income;*

*Model 3: Model 2 adjusted with exercise, cigarette smoking status, biomass fuel, alcohol consumption, red meat consumption*

*AIC* Akaike information criterion, *CI* Confidence interval, *FBG* Fasting blood glucose, *HDL-c* High-density lipoprotein cholesterol, *MetS* Metabolic syndrome, *OR* Odd ratio, *TG* Triglyceride

**Fig. 1:**
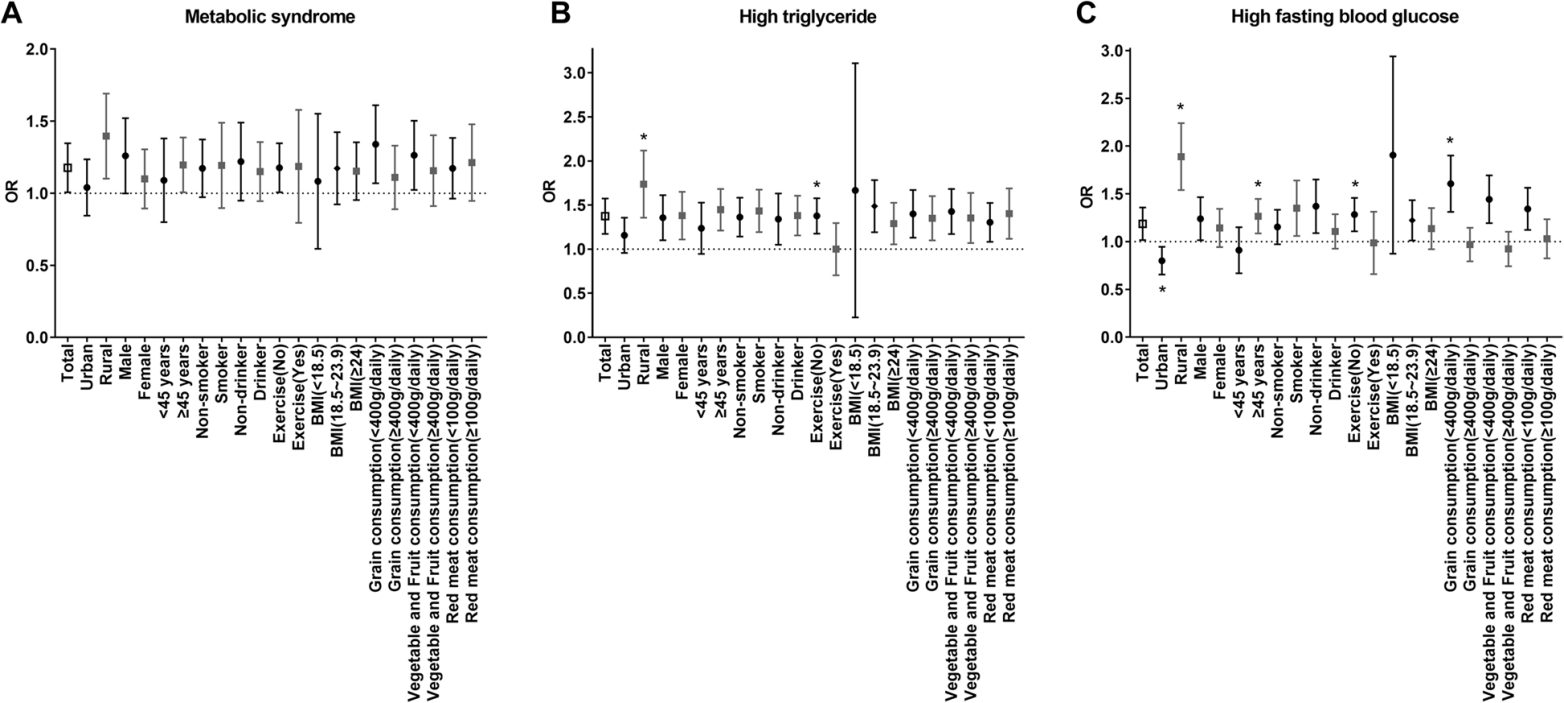
Associations of long-term PM_2.5_ exposure with MetS, high TG and high FBG in different stratum (*represents the *p* value for interaction with significance). **A**) Metabolic syndrome, **B**) High triglyceride, **C**) High fasting blood glucose

Table 5 shows the subgroup analysis by the region, sex, age, cigarette smoking, alcohol consumption, exercise, BMI, grain consumption, vegetable and fruit consumption and red meat consumption (Table 5, Fig. 1). We did not find statistically significant interactions between PM_2.5_ and the aforementioned variables for MetS. We observed stronger associations between PM_2.5_ and high TG levels in subgroups who took less exercise, living in rural area, with statistically significant interactions (*P*_interaction_ < 0.05). In addition, each 10 μg/m^3^ increase in two-year mean exposure to PM_2.5_ was associated with 87%, 26%, 59% and 28% and higher risk of high FBG among subgroups living in rural area, ≥ 45 years old, having < 400 g/daily grain intake and less exercise, with statistically significant interactions among these groups (*P*_interaction_ < 0.05).

**Table 5:** Subgroup analysis of the association between per two-year mean 10-μg/m^3^ increase in PM_2.5_ and metabolic syndrome, high triglyceride and high fasting blood among adults and elderly

**Variable**	**MetS**	*P*_*inter*_	**High TG**	*P*_*inter*_	**High FBG**	*P*_*inter*_
	**OR (95%CI)**		**OR (95%CI)**		**OR (95%CI)**	
**Region**		0.054		0.004*		< 0.001^*^
Urban (*n* = 3613)	1.03 (0.85, 1.24)		1.15 (0.96, 1.36)		0.79 (0.66, 0.95) ^*^	
Rural (*n* = 3015)	1.38 (1.11, 1.70) ^*^		1.71 (1.37, 2.13) ^*^		1.87 (1.55, 2.25) ^*^	
**Sex**		0.275		0.848		0.517
Men (*n* = 2955)	1.24 (1.01, 1.53) ^*^		1.34 (1.11, 1.62) ^*^		1.23 (1.02, 1.47) ^*^	
Women (*n* = 3673)	1.09 (0.90, 1.31)		1.36 (1.12, 1.66) ^*^		1.13 (0.95, 1.35)	
**Age**		0.412		0.083		0.049^*^
< 45 years(*n* = 2316)	1.07 (0.81, 1.39)		1.21 (0.96, 1.54)		0.89 (0.68, 1.16)	
≥ 45 years(*n* = 4312)	1.19 (1.01, 1.39) ^*^		1.43 (1.22, 1.69) ^*^		1.26 (1.09, 1.45) ^*^	
**Cigarette smoking**		0.793		0.637		0.081
Nonsmoker(*n* = 4428)	1.16 (0.98, 1.38)		1.35 (1.15, 1.59) ^*^		1.14 (0.98, 1.34)	
Smoker(*n* = 2200)	1.17 (0.91, 1.50)		1.42 (1.20, 1.68) ^*^		1.33 (1.07, 1.65) ^*^	
**Alcohol consumption**		0.156		0.195		0.261
Non-drinker (*n* = 3929)	1.20 (0.96, 1.50)		1.32 (1.06, 1.64) ^*^		1.35 (1.10, 1.66) ^*^	
Drinker (*n* = 2699)	1.14 (0.95, 1.36)		1.37 (1.16, 1.61) ^*^		1.10 (0.93, 1.29)	
**Exercise**		0.269		0.015^*^		0.040^*^
No (*n* = 5479)	1.17 (1.01, 1.35) ^*^		1.37 (1.18, 1.58) ^*^		1.28 (1.11, 1.46) ^*^	
Yes (*n* = 1149)	1.14 (0.82, 1.60) ^*^		0.97 (0.72, 1.31) ^*^		0.95 (0.68, 1.33)	
**BMI**		0.795		0.681		0.349
Underweight(*n* = 504)	1.02 (0.65, 1.58)		1.25 (0.48, 3.27)		1.72 (0.98, 3.02)	
Normal (*n* = 3741)	1.16 (0.93, 1.43)		1.47 (1.20, 1.79) ^*^		1.21 (1.02, 1.44) ^*^	
Over weight/Obese (*n* = 2383)	1.14 (0.96, 1.36)		1.28 (1.06, 1.53) ^*^		1.12 (0.93, 1.36)	
**Grain consumption**		0.713		0.897		< 0.001^*^
< 400 g/daily(*n* = 3232)	1.32 (1.08, 1.62) ^*^		1.38 (1.14, 1.68) ^*^		1.59 (1.32, 1.91) ^*^	
≥ 400 g/daily (*n* = 3396)	1.09 (0.90, 1.34)		1.33 (1.11, 1.61) ^*^		0.96 (0.80, 1.15)	
**Vegetable and Fruit consumption**		0.979		0.869		0.419
< 400 g/daily(*n *= 3858)	1.25 (1.03, 1.51) ^*^		1.41 (1.18, 1.69) ^*^		1.43 (1.20, 1.70) ^*^	
≥ 400 g/daily (*n* = 2770)	1.14 (0.92, 1.41)		1.36 (1.10, 1.66) ^*^		0.91 (0.75, 1.11)	
**Red Meat consumption**		0.312		0.332		0.860
< 100 g/daily (*n* = 3970)	1.16 (0.97, 1.39)		1.29 (1.09, 1.53) ^*^		1.33 (1.13, 1.57) ^*^	
≥ 100 g/daily (*n* = 2658)	1.19 (0.96, 1.49)		1.38 (1.13, 1.70) ^*^		1.02 (0.83, 1.24)	

*AIC* Akaike information criterion, *BMI* Body-mass index, *CI* Confidence interval, *FBG* Fasting blood glucose, *HDL-C* High-density lipoprotein cholesterol, *MetS* Metabolic syndrome, *OR* Odd ratio, *TG* Triglyceride

Adjusted with age, sex, education, marital status, body mass index, household income, exercise, cigarette smoking status, biomass fuel, alcohol consumption, red meat consumption

^∗^: *P* < 0.05

## Discussion

Understanding the impacts of long-term exposure to ambient PM_2.5_ on MetS is crucial, because 25.5% of the population had MetS in the studied regions of southern China. This study was conducted to elucidate the key research question regarding whether exposure to ambient PM_2.5_ would increase the risk of having MetS and confer a detrimental impact on its specific components in Guangdong province. Information regarding the associations between PM_2.5_ and the prevalence of MetS with its specific components in China remains scarce. Reassuringly, we found that long-term exposure to ambient PM_2.5_ pollution was significantly associated with an increased risk of MetS. In addition, long-term exposure to PM_2.5_ increased the risk of high TG and high FBG. Furthermore, the participants living in rural area, aged greater than 45 years, having less exercises and < 400 g/daily grain intake were more susceptible to the adverse effects of ambient PM_2.5_ exposure.

Although previous studies and the current study were conducted in different geographical areas, with differences in the population characteristics, pollutant concentrations or sources, exposure duration and exposure measurement, it is worth mentioning that positive associations of long-term ambient PM_2.5_ pollution exposure with MetS remained consistent and that the magnitudes of the effect estimates observed in these studies were comparable. The normative aging study in New York [17] and a cross-sectional study in China [27] found that 10 μg/m^3^ increase in ambient PM_2.5_ was associated with a 10% to 31% higher risk of MetS among children, adolescents and elderly population. A nationwide population-based cohort study in Korea showed that each 10 µg/m^3^ increase in one-year averaged concentration of PM_2.5_ was associated with a 7% higher risk of MetS in adults [25]. Likewise, the Chinese health study found that each 10 μg/m^3^ increase in the long-term exposure to PM_2.5_ was associated with 5% higher risk of MetS in 15,477 adults from 33 communities in northeast China [28]. We have detected the largest magnitude of effect estimates of the association between PM_2.5_ and MetS in adults. Compared with other heavy industry cities in northeast China, higher risk of total, cardiovascular and respiratory mortality was found in Guangzhou, where the concentration of PM was relatively low [31]. The relatively high concentration of the toxic components (e.g. PBDEs) in PM_2.5_ detected in southern China [32, 33] might help explain the paradoxically larger effect estimates of the association between PM and total/cardiovascular/respiratory disease mortality and MetS, in the scenario of the lower concentration of PM in Guangdong.

Regarding the complexity of metabolic alterations that constitute MetS, many studies have investigated the association between long- and short-term exposure of PM_2.5_ and its specific components [15, 17, 21, 42–45]. Several population-based studies have reported harmful effects of ambient PM_2.5_ on FBG, yet the results were inconsistent. Though Alderete et al. did not identify a statistically significant association between long-term exposure to PM_2.5_ and FBG in Los Angeles Latino children [21], several other studies investigating the harmful effects of PM_2.5_ on FBG has supported our findings in different population [15, 42, 43]. The Normative Aging Study found that exposure to high levels of PM_2.5_ within 28 days was associated with an increased level of FBG [43]. A cross-sectional study revealed a positive association between exposure to PM_2.5_ and increased FBG among primary school children in China [15]. Few studies have investigated the relationship between PM_2.5_ and high TG. We are awared of only three studies which were conducted in specific populations or yielded different results from this study. Similar to the results from 587 elderly individuals in the US [17] and 73,117 subjects with known CVDs and risk factors in southern Israel [44], we have identified the adverse impact of PM_2.5_ on TG. However, none of the significant association was found in the population-based cross-sectional study conducted in northeast China [45]. Similar to the results of Wallwork RS et al. [17], we did not reveal a significant association between PM_2.5_ and abdominal obesity, low HDL-c and hypertension, which are the essential components of MetS that are often presented as the underlying and/or preceding other components [46] and cardiovascular events [47, 48]. PM_2.5_ might activate the metabolic mechanisms such as inflammation, which might increase the risk of developing elevated FBG and hypertriglyceridemia without substantially increasing the risk of abdominal obesity, low HDL-c or hypertension.

As seen in other air pollutant studies, the health effects shown in our study were relatively small. However, regarding the broad extent of the exposed population and the continuous nature of exposure, health implications of ambient PM_2.5_ exposures should be considered at the population level rather than at the individual level [49, 50]. Metabolic risk factors have long been hypothesized as the mediators between air pollutants and CVDs [45, 51, 52]. A previous study showed that participants with an existing metabolic risk factor had a higher risk of CVDs than those without [45]. The results of high TG and high FBG attributed to PM_2.5_ based on our analyses may help provide the evidence to support these hypotheses. In addition, MetS, high FBG and TG can be translated into adverse health outcomes of CVDs and diabetes mellitus [4, 5]. Participants with type 2 diabetes and hypertriglyceridemia may be more susceptible to the cardiovascular effects of PM_2.5_ than those without cardiometabolic risk factors. Small differences in the glucose/TG control within the normal range could be translated into the clinically meaningful variation in CVDs and diabetes mellitus risk [53]. These metabolic associations may represent the intermediate factors that help explain the detrimental effect of increased exposure to PM_2.5_ on CVDs and diabetes mellitus morbidity and mortality. Nevertheless, our findings were not unexpected because air pollution exposure and metabolic risk factors have been closely associated with the heightened inflammatory responses, which is implicated in the development of CVD [52]. Thus, participants with high TG and high FBG might be more susceptible to the detrimental effects of PM_2.5_, which could help interpret a higher CVD prevalence.

There were limitations regarding the study design and data interpretation. The causality between ambient PM_2.5_ exposure and MetS and its components cannot be confirmed owning to the cross-sectional study design. Second, data on the secondary MetS diseases were also not fully collected. Although we have excluded participants with CVDs, other diseases including hyperlipidemia and renal hypertension were not available, which might have influenced on the results. Third, the information on multiple food intake was limited regarding the importance of such variable on the etiology of MetS. Furthermore, there could be interactions between PM_2.5_ and multiple indoor air pollutants (e.g., mold, household fuels, allergens, tobacco smoke, cooking, furniture, paints, cleaning agents) [54], which cannot be readily disentangled.

However, our findings remain robust. We conducted the LUR model to determine PM_2.5_ exposure at a specific address to safeguard the accuracy of the exposure assessment. Additionally, our association analyses were based on multiple models, with the results not being materially altered. Because the long-term health risk of TG and FBG may be important predictors for future risks of CVDs and diabetes mellitus, efforts should be endeavored to minimize the concentration and exposure to PM_2.5_ pollution.

## Conclusion

In conclusion, this study adds to the comprehensive evidence of the association between long-term exposure to PM_2.5_ and MetS. Dyslipidemia especially high triglyceride and FBG impairment is strongly associated with PM_2.5_ levels. However, further prospective studies are needed to confirm our findings.

## Supplementary Information

**Additional file 1.**

## Abbreviations

CVDs: Cardiovascular diseases
PM_2.5_: Particulate matter ≤ 2.5 µm
PM_10_: Particulate matter < 10 µm
SO_2_: Sulfur dioxide
NO_2_: Nitrogen dioxide
O_3_: Ozone
MetS: Metabolic syndrome
FBG: Fasting blood glucose
TG: Triglyceride
HDL-c: High-density lipoprotein cholesterol
LUR: Land-use regression
AIC: Akaike information criterion
OR: Odd ratio
CI: Confidence interval
PBDEs: Polybrominated diphenyl ethers
RMSE: Root mean square error
BMI: Body-mass index
ICC: Intraclass correlation coefficient
VIF: Variance inflation factor
WHO: World Health Organization
