# Supplementary material for: Effects of long-term PM2.5 exposure on metabolic syndrome among adults and elderly in Guangdong, China
Source: Environ Health. 2022 Sep 10;21:84. doi: 10.1186/s12940-022-00888-2 (PMC9464395; doi:10.1186/s12940-022-00888-2)
Supplement: Supplementary file 1 — Additional file 1. [file 12940_2022_888_MOESM1_ESM.docx]

**E-Table 1. Type and definition of the variable in the questionnaire.**

| **Variable** | **Types of variables** | **Definition** |
| --- | --- | --- |
| **Age**^1-2^ | quantitative variable | Years of age; date of interview minus date of birth |
| **Sex**^1-2^ | qualitative variable |  |
| Male |  | Male |
| Female |  | Female |
| **Race**^1-2^ | qualitative variable |  |
| Han |  | Han ethnicity |
| Minority |  | Except for Han ethnicity, other ethnicity in China |
| **Region**^1-2^ | qualitative variable |  |
| Urban |  | Urban regions defined by the administrative regions in China |
| Rural |  | Rural regions defined by the administrative regions in China |
| **Occupation**^1-2^ | qualitative variable |  |
| Physical work |  | Occupation includes production staff of agriculture, forestry, animal husbandry, fishery and marine industry; production and transportation equipment operators; soldiers and domestic workers |
| Mental work |  | Occupation includes students, unemployed person, civil servant, retirees and professionals |
| **Educational level**^1-2^ | qualitative variable |  |
| None |  | Without any education experience |
| Primary school education |  | Primary school education |
| Middle school education |  | Middle school education |
| University education or higher |  | University education or higher |
| **Marriage status**^1-2^ | qualitative variable |  |
| Unmarried |  | Unmarried |
| Married |  | Married |
| Widowed or divorced |  | Widowed or divorced |
| **Household income (×1000 RMB)**^1-2^ | qualitative variable |  |
| < 30 |  | Annual income with < 30,000 RMB |
| 30≤Household income <50 |  | Annual income ranging from 30,000 to 50,000 RMB |
| 50≤Household income<100 |  | Annual income ranging from 50,000 to 100,000 RMB |
| 100≤Household income<200 |  | Annual income ranging from 100,000 to 200,000 RMB |
| ≥200 |  | Annual income with ≥200,000 RMB |
| Refuse to answer or don't know |  | - |
| **W****eight change in the past 12 months**^1-2^ |  |  |
| An increase in > 2.5 kg |  | Increase in > 2.5 kg |
| Unchanged (< 2.5 kg) |  | Unchanged (< 2.5 kg) |
| A decease in > 2.5 kg |  | Decease in > 2.5 kg |
| Unclear |  | Unclear |
| **Family history of diabetes mellitus**^1-2^ | qualitative variable |  |
| No |  | No relative has diabetes mellitus |
| Yes |  | Relatives including grandfathers, grandmothers, fathers, mothers, brothers and sisters have diabetes mellitus |
| **Alcohol consumption**^1-2^ | qualitative variable |  |
| No |  | No drinking alcohol in the past 12 months |
| Yes |  | Drinking alcohol in the past 12 months |
| **Exercise**^1-2^ | qualitative variable |  |
| No |  | No physical exercise |
| Yes |  | Taking physical exercise for more than 10 minutes at least once a week |
| **Passive smoking**^1-2^ | qualitative variable |  |
| No |  | No exposure to secondhand smoke |
| Yes |  | Exposure to secondhand smoke every day or 4-6 day every week or 1-3 day every week. |
| **Cigarette smoking**^3^ | qualitative variable |  |
| Nonsmoker |  | Never smokers |
| Smoker |  | Ex- and current smokers who actively smoked cigarettes every day or occasionally |
| **Biomass fuel**^3^ | qualitative variable |  |
| No |  | Household cooking fuels are natural gas, solar and//or others |
| Yes |  | Household cooking fuel are biomass fuel (wood, grass, crop residues, and animal dung) and/or coal fuels (including coal and kerosene) |
| **Grain consumption**^4^ | quantitative variable | Daily intake of various grains, including rice and its products, wheat and its products, millet, etc. |
| **Vegetable and Fruit consumption**^4^ | quantitative variable | Daily intake of various types of fresh, unprocessed vegetables and fruits |
| **Red Meat consumption**^4^ | quantitative variable | Daily intake of various types of unprocessed fresh or frozen livestock meat, including beef, pork, lamb, etc. |
| **BMI category**^5^ | qualitative variable |  |
| Under weight |  | BMI<18.5kg/m^2^ |
| Normal |  | 18.5≤BMI< 24 kg/m^2^ |
| Overweight/ Obese |  | BMI≥24 kg/m^2^ |
| **Central obesity** |  | Elevated waist circumference: ≥90cm for males; ≥80cm for females |
| **High triglyceride** |  | Elevated TG levels≥1.7 mmol/l (150 mg/dl) |
| **Low high-density lipoprotein cholesterol** |  | Decreased HDL-C levels: <1.0 mmol/l (40 mg/dl) for males;<1.3 mmol/l (50 mg/dl) for females |
| **Hypertension** |  | Elevated blood pressure (SBP ≥130 or DBP ≥85 mmHg) |
| **High fasting blood glucose** |  | Elevated FBG levels [FBG≥5.6 mmol/l (100 mg/dl)] |
| **Metabolic syndrome** |  | Participants were considered to have MetS if they meet any three of the five following conditions (1):Elevated TG levels: ≥1.7 mmol/l (150 mg/dl); (2) Decreased HDL-C levels: <1.0 mmol/l (40 mg/dl) for males;<1.3 mmol/l (50 mg/dl) for females; (3) Elevated blood pressure (SBP ≥130 or DBP ≥85 mmHg); (4) Elevated FBG levels [FBG ≥5.6 mmol/l (100 mg/dl)]; (5) Elevated waist circumference: ≥90cm for males; ≥80cm for females. |

Reference：

1. Wang B, He M, Chao A, Engelgau MM, Saraiya M, Wang L, Wang L. Cervical Cancer Screening Among Adult Women in China, 2010. Oncologist. 2015 Jun;20(6):627-34.
2. Wang L, Peng W, Zhao Z, Zhang M, Shi Z, Song Z, Zhang X, Li C, Huang Z, Sun X, Wang L, Zhou M, Wu J, Wang Y. Prevalence and Treatment of Diabetes in China, 2013-2018. JAMA. 2021 Dec 28;326(24):2498-2506.
3. Zheng XY, Tang SL, Guan WJ, Ma SL, Li C, Xu YJ, Meng RL, Lin LF. Exposure to biomass fuel is associated with high blood pressure and fasting blood glucose impairment in females in southern rural China. Environ Res. 2021 Aug;199:111072.
4. Cai L, Wang S, Gao P, Shen X, Jalaludin B, Bloom MS, Wang Q, Bao J, Zeng X, Gui Z, Chen Y, Huang C. Effects of ambient particulate matter on fasting blood glucose among primary school children in Guangzhou, China. Environ Res. 2019 Sep;176:108541.
